# Causal associations of white blood cell count and sudden sensorineural hearing loss: a bidirectional and multivariable Mendelian randomization study

**DOI:** 10.3389/fneur.2024.1387244

**Published:** 2024-09-27

**Authors:** Ling Zhang, Jialei Chen, Shixun Zhong, Jing Luo, Ping Tang

**Affiliations:** ^1^Department of Otorhinolaryngology, The People’s Hospital of Yubei District of Chongqing, Chongqing, China; ^2^Department of Otolaryngology, The First Affiliated Hospital of Chongqing Medical University, Chongqing, China; ^3^Department of Pathology and Pathophysiology, Chongqing Medical University, Chongqing, China; ^4^Molecular Medicine and Cancer Research Center, College of Basic Medical Sciences, Chongqing Medical University, Chongqing, China; ^5^Department of Neurology, The First Affiliated Hospital of Chongqing Medical University, Chongqing, China; ^6^Department of Pathology, Chongqing Medical University, Chongqing, China

**Keywords:** sudden sensorineural hearing loss, lymphocyte cell count, monocyte cell count, neutrophil cell count, Mendelian randomization

## Abstract

**Background:**

Numerous compelling observational studies have demonstrated a plausible correlation between the white blood cell count and the susceptibility to sudden sensorineural hearing loss (SSNHL). Nevertheless, the exact causal relationship between these two factors remains ambiguous. The objective of our study was to assess the causal impact of white blood cell count on sudden sensorineural hearing loss through the implementation of a bidirectional and multivariable Mendelian randomization (MR) methodology.

**Methods:**

Genetic data pertaining to white blood cell count were acquired from the Blood Cell Consortium, encompassing a total of 563,946 subjects. Concurrently, summary data on sudden sensorineural hearing loss were sourced from a Genome-Wide Association Study (GWAS), involving 196,592 participants, comprising 1,491 cases and 195,101 controls. The primary method employed for MR analysis was the Inverse Variance Weighted method (IVW), while sensitivity analysis utilized the Weighted Median method, MR-Egger method, and MR-PRESSO method.

**Results:**

In IVW method, genetically predicted elevated lymphocyte cell count demonstrates an effective reduction in the risk of sudden sensorineural hearing loss (odds ratio = 0.747, 95% CI = 0.565–0.987, *p* = 0.04). These findings remain consistent in multivariate MR analysis, even after adjusting for monocyte cell count and neutrophil cell count levels (odds ratio = 0.929, 95% CI = 0.867–0.995, *p* = 0.036). However, there is no discernible evidence supporting a direct causal relationship between monocyte cell count and neutrophil cell count levels and the occurrence of SSNHL.

**Conclusion:**

Within the normal range, higher lymphocyte cell count levels exhibit a potential protective effect against SSNHL. Meanwhile, no direct causal relationship are identified between monocyte cell count and neutrophil cell count levels and the risk of SSNHL.

## 1 Introduction

Sudden sensorineural hearing loss (SSNHL) represents an otological emergency characterized by an unknown etiology influenced by diverse factors. It is defined as the abrupt onset of unexplained sensorineural hearing loss within a 72 h timeframe, involving a hearing loss exceeding 30 dB across a minimum of three consecutive frequencies (1). Epidemiological studies indicate that in industrialized nations, the annual incidence rate of SSNHL ranges from 5 to 400 cases per 100,000 individuals (2). SSNHL typically manifests as a unilateral, isolated condition, displaying distinct clinical characteristics in terms of hearing loss severity, accompanying symptoms, and prognosis. Numerous investigations suggest a close association between vascular dysfunction (3), infectious diseases (4), autoimmune conditions (5), and other factors with the onset and progression of SSNHL, signifying its likely multifactorial origin rather than a singular cause. The global incidence of SSNHL is escalating rapidly, and as of yet, no proven or universally recommended treatment exists (6). Consequently, otologists face an imperative need to identify biomarkers for predicting the occurrence and progression of SSNHL. This is essential for the development of more effective prevention and treatment strategies tailored to address this challenging condition.

In recent times, the etiological investigation of sudden sensorineural hearing loss has prominently centered around chronic inflammation (7). The cochlea’s blood supply predominantly relies on a single cochlear artery, rendering it susceptible to damage from ischemia and hypoxia. Given this delicate anatomical condition, chronic inflammation induced by various factors may precipitate vascular dysfunction and an immune response in the cochlea, ultimately resulting in cochlear ischemia and injury (8). White blood cells and their constituents serve as cost-effective and valuable inflammatory markers in clinical practice. White blood cell count are widely employed as predictive markers for various diseases, such as diabetes (9), kidney disease (10), and cardiovascular conditions (11). Elevated white blood cell count are frequently observed in patients with SSNHL. However, it is essential to note that these findings are derived from clinical observations, introducing the potential for selection bias, confounding factors, and the risk of reverse causality. Consequently, the causal relationship between white blood cell count and SSNHL remains an open question. Unraveling this causal connection is pivotal in formulating effective prevention and treatment strategies for SSNHL.

Mendelian randomization employs genetic variants as instrumental variables to explore the causal relationships between disease-related risk factors (12). This emerging epidemiological methodology effectively mitigates potential confounding factors and interferences, enabling the derivation of more robust causal conclusions compared to traditional observational studies (13). Previous MR analyses have successfully elucidated the causal connections between thyroid hormones (2), blood lipids (14), and sudden sensorineural hearing loss. The present study employs Bidirectional and multivariate MR analysis to assess the association between genetically predicted white blood cell count and the corresponding SSNHL risk. Three specific white blood cell count of interest—lymphocyte cell count, neutrophil cell count, and monocyte cell count—have been identified, demonstrating associations with infection risk and detectable through genetic instruments. This endeavor aims to offer novel perspectives and insights into the etiology of SSNHL.

## 2 Method

### 2.1 Study design

**Figure 1:**
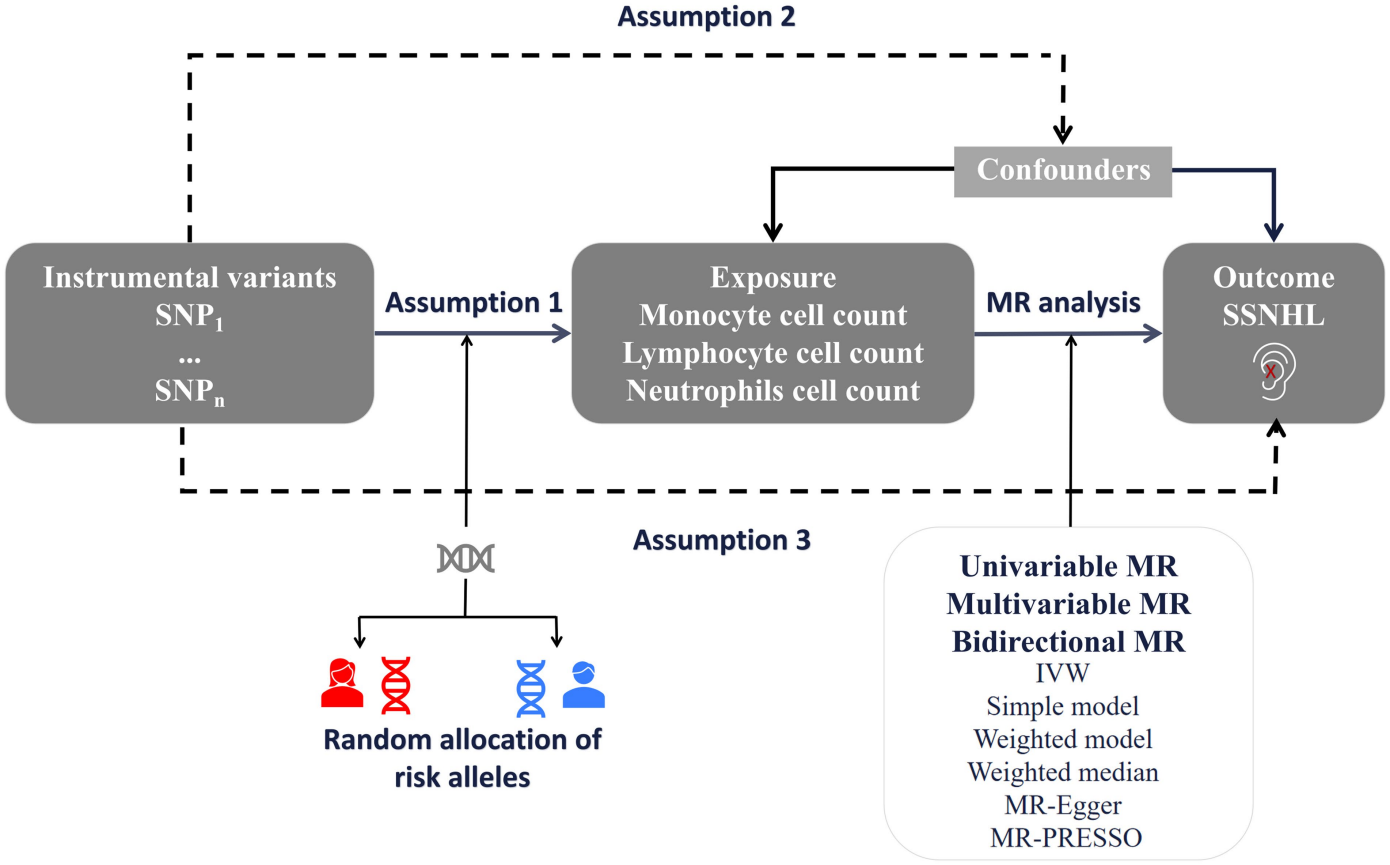
A framework design for bidirectional and multivariable MR analyses. Assumption 1: genetic variants are associated with exposure. Assumption 2: genetic variants are not associated with any know or unknown confounders. Assumption 3: Genetic variants should only affect the risk of outcome through exposure. SSNHL, sudden sensorineural hearing loss; MR, Mendelian randomization.

We conducted a bidirectional and multivariate MR study utilizing Genome-Wide Association Study (GWAS) data for both white blood cell count and sudden sensorineural hearing loss. To minimize population stratification bias, both the exposure and outcome cohorts were confined to individuals of European ancestry. The robust MR design hinges on three fundamental assumptions: (1) The correlation hypothesis posits a strong correlation between genetic variation and exposure factors, in this case, white blood cell count. (2) The independence hypothesis assumes that gene variation is independent of confounding factors that might influence both exposure and outcome. (3) The exclusivity hypothesis suggests that genetic variation impacts the outcome solely through exposure and not through alternative pathways, specifically SSNHL (15). Figure 1 offers an overview of the design employed in the bidirectional and multivariate MR study of white blood cell count and SSNHL. Given that this study involved the reanalysis of previously published data, no additional ethical approval was deemed necessary.

### 2.2 GWAS data of white blood cell count

The genetic data for white blood cell count were sourced from the Blood Cell Consortium (16), encompassing a total of 563,946 subjects included in this study. These data are publicly accessible and downloadable from Genome-Wide Association Study websites. Their respective GWAS IDs are ieu-b-31 (monocyte cell count), ieu-b-32 (lymphocyte cell count), and ieu-b-34 (neutrophil cell count).

### 2.3 GWAS data of SSNHL

Genetic data related to sudden sensorineural hearing loss were acquired from the publicly accessible Genome-Wide Association Study (GWAS) database, specifically identified with the entry number “finn-b-H8_HL_IDIOP.” The study involved a total of 196,592 participants, comprising 1,491 cases and 195,101 controls (17).

### 2.4 Instrumental variable selection

Based on the GWAS results for white blood count, a meticulous screening of single nucleotide polymorphisms (SNPs) closely associated with white blood count and achieving genome-wide significance (*p* < 5 × 10^−8^) was conducted (16). However, during the reverse MR analysis, considering the limited sample size and number of SNPs, we loosened the association threshold to select SNPs related to SSNHL, setting the significance level at *p* < 5 × 10^−6^ (17). These selected SNPs were subsequently utilized as IVs in MR analysis (18). The criteria for IVs selection in MR analysis were as follows: (1) To mitigate estimation bias stemming from weak IVs, the equation F = (*R*^2^ × (*n*−2))/(1−R2) was employed to evaluate the correlation between instrument strength and exposure. A significant correlation was considered when *F* ≥ 10. The estimated *R*^2^ for IVs was calculated using the equation 2EAF (1−EAF)*β^2^, where EAF represents the frequency of the effector allele, and β represents the estimated genetic effect on exposure factors. (2) To address the impact of linkage disequilibrium, efforts were made to ensure that the r^2^ value was less than 0.001 at a distance of 10 MB, and palindromic SNPs with moderate allele frequencies were excluded. (3) In adherence to the exclusivity hypothesis (IV variants only affecting SSNHL through white blood cell count), all SNPs associated with hearing loss (*p* < 1 × 10^−5^) were excluded from each analysis (19, 20). The PhenoScanner database^1^ was utilized to eliminate all known phenotypes associated with any genetic tools considered in our analysis (21). In Supplementary Table S1, a comprehensive summary is provided, detailing the relationship between exposure, SNPs, and their associations with outcomes.

### 2.5 Univariate Mendelian randomization analysis

Utilizing the IVW method as the primary analysis, we employed a range of complementary Mendelian randomization tests to rigorously examine causal effects and correct for the impact of horizontal multiplicity (22). These included the weighted median method, the simple mode method, the MR-Egger regression method, and the MR-pleiosis residuals and outliers method (MR-PRESSO) (23–25). In essence, the IVW method effectively combines the causal effects of individual single SNPs. However, it is crucial to emphasize that this method yields unbiased estimates of causal effects only under the condition that all SNPs are devoid of invalid IVs and horizontal pleiotropy. In response to this concern, additional sensitivity analyses were performed using MR Egger and weighted median as complementary methods to IVW. The MR-Egger method serves to assess the presence of horizontal pleiotropic effects among all SNPs through the intercept, providing a reliable and unbiased evaluation of causality. A *p*-value below 0.05 indicates the existence of horizontal pleiotropy. The weighted median method, relying on the median effect of all available genetic tools, ensures consistency in potential causality if at least half of the genetic variation adheres to assumptions. The MR-PRESSO method, designed to identify and eliminate outliers, generates relatively unbiased estimates while detecting potential horizontal pleiotropic effects through global testing. Cochran’s Q test was applied to assess SNP heterogeneity, with a *p*-value >0.05 for Cochran’s Q test indicating no heterogeneity. Additionally, a leave-one-out analysis was conducted, systematically removing one SNP at a time to evaluate whether bias in MR estimates was driven by a single SNP. Reverse MR analysis explored the possibility of sudden sensorineural hearing loss acting as a risk factor for white blood count. To ensure the validity of bidirectional MR, genetic instruments exposed in bidirectional analysis (white blood count or SSNHL) were scrutinized for independence, revealing no overlapping SNPs or SNPs in high linkage disequilibrium. Given that genotypes are determined at conception in accordance with Mendel’s laws of segregation, the likelihood of reverse causation is significantly diminished (26).

### 2.6 Multivariable Mendelian randomization analysis

Multivariate Mendelian randomization operates analogously to independently assessing the effects of various intervention modalities in a randomized controlled trial. In this methodology, genetic instruments may exhibit associations with multiple risk factors, provided they meet the prerequisite of being equivalent instrumental variables (27). Given the close genetic correlation observed between monocyte cell count, lymphocyte cell count, and neutrophil cell count, coupled with their analogous associations with SSNHL in observational studies. In this analysis, we included all instrumental variables for monocyte cell count, lymphocyte cell count, and neutrophil cell count to assess their independent impacts on SSNHL. The SNPs employed in multivariate MR analysis were derived from combinations of instrumental variables identified in univariate MR analyses for each exposure (28). Statistical significance in estimating the causal effect of exposure was determined with *p*-values less than 0.05. All statistical analyses were carried out using the R package “TwoSampleMR2 (version 0.5.6)” and “Mendelian Randomization” (version 0.5.1) in R (version 4.2.1). For a more detailed description, please refer to the following link^2^ (29).

## 3 Result

### 3.1 Univariate MR analysis of the causal relationship between white blood cell count and SSNHL

**Figure 2:**
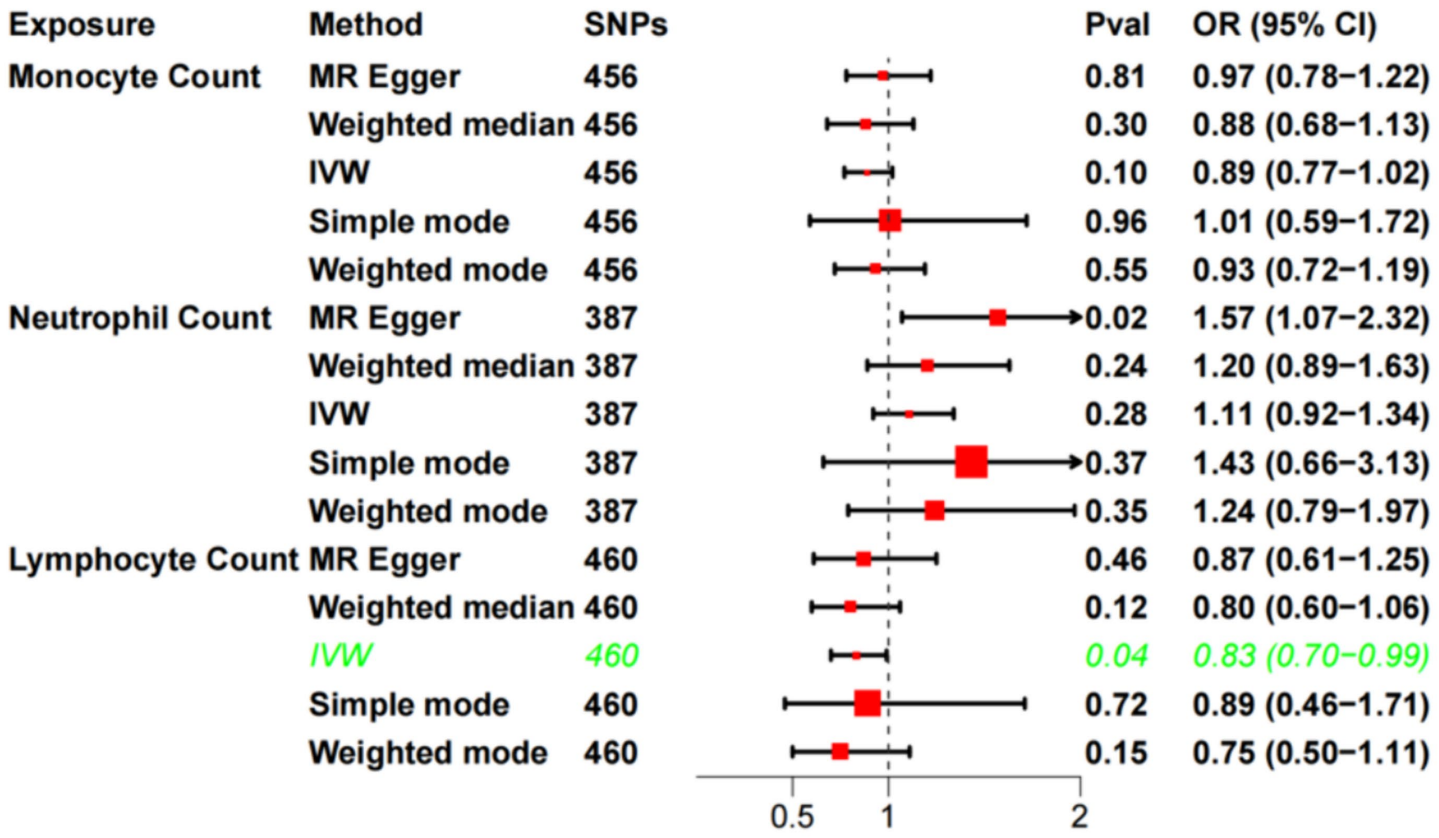
Univariate MR analysis of the causal relationship between white blood cell count and SSHNL.

**Figure 3:**
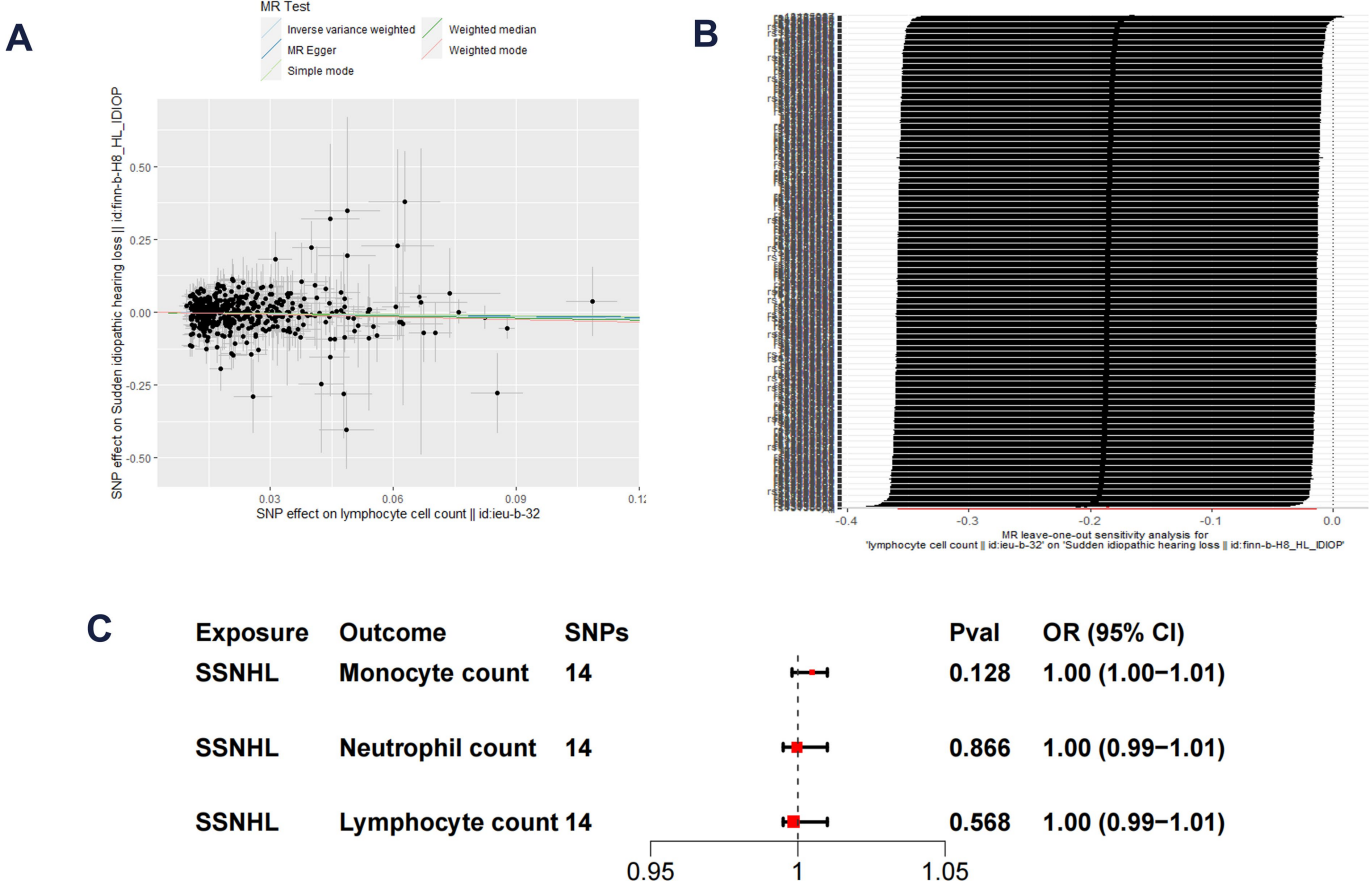
**(A)** Scatter plot demonstrate the effect of each lymphocyte cell count-associated genetic variant and SSNHL on the log-odds scale. **(B)** Leave-one-out plots for the MR analyses of lymphocyte cell count and SSNHL. **(C)** Reverse MR analysis of SSNHL and white blood cell count.

The F-statistics for each SNP included in the analysis exceeded 10 (Supplementary Table S1). The results of the univariate MR analysis, after assessing and removing SNPs associated with confounding, are depicted in Figure 2. The MR analysis utilizing the IVW method revealed a significant causal relationship between lymphocyte cell count and the risk of sudden sensorineural hearing loss (OR = 0.83, 95%CI = 0.70–0.99, *p* = 0.04). Similarly, risk estimates from MR-Egger regression and weighted median methods exhibited similar trends, although these associations did not reach statistical significance (Figure 3A). *p*-values obtained from the Cochran Q tests for MR-Egger (Cochrane’s Q = 438.6, *p* = 0.73) and IVW (Cochrane’s Q = 438.7, *p* = 0.74) were greater than 0.05, indicating no heterogeneity in the results. The global test for MR-PRESSO (P Global Test = 0.71) and Egger_intercept (−0.0013) and the *p* values derived from Egger intercepts (0.75) indicated that no anomalous instrumental variables contributed to the effect of multiplicity in the overall MR estimates. Leave-one-out sensitivity analyses affirmed the robustness of the conclusion (Figure 3B). However, no evidence supporting a causal relationship was found between monocyte cell count (IVW, OR = 0.89, 95% CI = 0.77–1.02, *p* = 0.10) and neutrophil cell count (IVW, OR = 1.11, 95% CI = 0.92–1.34, *p* = 0.28) and SSNHL. Finally, a reverse MR analysis was performed to evaluate the causal effect of SSNHL on white blood cell count. After applying the aforementioned criteria, 14 SNPs significantly associated with SSNHL were identified (Supplementary Table S1). In our reverse MR analysis using the IVW method, no significant evidence supporting a causal relationship between SSNHL and the risk of white blood count levels was found (Figure 3C).

### 3.2 Multivariate MR analysis of the causal relationship between white blood cell count and SSNHL

**Figure 4:**
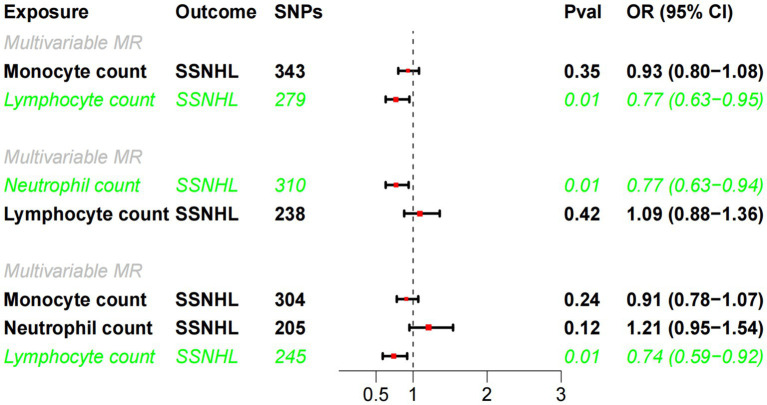
Multivariate MR analysis of the causal relationship between white blood cell count and SSHNL.

Building upon the robust correlation observed in observational studies between lymphocyte cell count, monocyte cell count, and neutrophil cell count, we conducted multivariate MR analyses to investigate their independent impacts on SSNHL. The findings of the multivariate MR analysis demonstrated that even after adjusting for monocyte cell count and/or neutrophil cell count, results consistent with the univariate MR analysis were attained (Figure 4). Lymphocyte cell count exhibited a negative association with the risk of developing sudden sensorineural hearing loss.

## 4 Discussion

Comprehending the pathogenesis of a disease is a fundamental prerequisite for the effective treatment of patients. Nevertheless, the precise pathophysiological mechanisms underlying sudden sensorineural hearing loss remain elusive. It is postulated that SSNHL may arise from a combination of local and systemic factors, with thrombosis and infection considered the most common causes. Notably, Chinese and German guidelines attribute thrombosis as the principal pathophysiological feature of SSNHL (30). However, Weng (31) and Qiao (32) contested the thrombosis hypothesis, pointing out the absence of a gender-based incidence difference. Recent compelling evidence has significantly shifted focus towards the role of chronic inflammation in SSNHL. Studies indicate that chronic inflammation induced by bacteria or viruses can lead to microvascular damage and atherosclerosis. Given the cochlea’s unique blood supply, primarily reliant on a single labyrinthine artery without collateral circulation, these factors directly elevate the risk of cochlear ischemia. The cochlear hair cells, characterized by high oxygen consumption, render the cochlea particularly susceptible to hypoxia, heightening sensitivity to alterations in blood circulation (8).

Biomarkers associated with inflammation in sudden sensorineural hearing loss patients encompassed elevated neutrophil, monocyte, and lymphocyte cell count, while composite markers linked to inflammation in these patients included heightened Neutrophil-to-Lymphocyte Ratio (NLR) and Monocyte-to-Lymphocyte Ratio (MLR) (33). Despite recent meta-analyses consistently indicating significantly higher neutrophil cell counts in SSNHL patients compared to the normal group (34), Sun (35) and Cao’s (36) study distinctly highlights that this elevation is confined to a specific subgroup of SSNHL patients. In our study, we found that monocyte cell count was not a risk factor for SSNHL. Koçak’s (37) study aligns with our findings, revealing no difference in monocyte cell count between the control and SSNHL groups. Interestingly, most studies demonstrate a significantly elevated monocyte-to-lymphocyte ratio in SSNHL patients compared to the control group (33, 34). Most studies have found a decrease in lymphocyte count in patients with SSNHL, with a high lymphocyte count being a protective factor against the risk of SSNHL, which aligns with our findings (35, 38, 39). To our knowledge, nearly all studies have reported a significantly higher NLR or (and) MLR in SSNHL patients than in the normal group. Except for lymphocyte cell count, no significant difference in any single inflammatory marker was identified in the prognosis of SSNHL (33). Current research on the etiology of SSNHL is predominantly focused on chronic inflammation (7). It is believed that chronic inflammation induced by bacteria or viruses can lead to microvascular damage, endothelial dysfunction, and atherosclerosis, thereby increasing the risk of cochlear ischemia (40–42). Lower lymphocyte counts are associated with an inflammatory response (43). Furthermore, an elevated NLR in the periphery indicates the occurrence of atherosclerosis and local microartery inflammation. In patients with SSNHL, a higher peripherally measured NLR suggests the presence of local microvascular inflammation, with the inflammation affecting the labyrinthine artery (44). These findings collectively indicate a profound association between the inflammatory response mediated by lymphocyte count and SSNHL (32). The varied conclusions across studies may arise from the categorization of SSNHL into at least four distinct subtypes, each with a unique pathogenic mechanism. Unfortunately, only a limited number of studies have conducted subgroup-specific analyses.

The design of this study offers notable advantages. Primarily, it leverages freely accessible GWAS data, thereby substantially reducing research costs. Nevertheless, it is essential to acknowledge several potential limitations in our study. Firstly, single blood inflammation markers are susceptible to various factors. In contrast, composite markers such as NLR and MLR are relatively stable, easily measurable, and cost-effective. Unfortunately, due to limitations in available pooled white blood cell count data, we were unable to conduct subgroup-specific Mendelian randomization analyses. Secondly, sudden sensorineural hearing loss comprises at least four subgroups with different pathogenic mechanisms. However, limitations in available SSNHL summary data hindered the performance of subgroup-specific MR analyses. Finally, the study population predominantly consisted of individuals of European descent, necessitating caution in interpreting the generalizability of our findings to other populations. Future research endeavors will encompass diverse populations and consider the impact of specific subgroups, thereby advancing our comprehension of the causal relationship between blood inflammatory indicators and SSNHL.

## Data Availability

The original contributions presented in the study are included in the article/Supplementary material, further inquiries can be directed to the corresponding authors.

## Footnotes

^1^http://www.phenoscanner.medschl.cam.ac.uk/phenoscanner

^2^https://mrcieu.github.io/TwoSampleMR/37
